# Methyl 1-methyl-1*H*-1,2,3-triazole-4-carboxyl­ate

**DOI:** 10.1107/S1600536810019173

**Published:** 2010-05-26

**Authors:** F. Nawaz Khan, K. Prabakaran, S. Mohana Roopan, Venkatesha R. Hathwar, Mehmet Akkurt

**Affiliations:** a Organic and Medicinal Chemistry Research Laboratory, Organic Chemistry Division, School of Advanced Sciences, VIT University, Vellore 632 014, Tamil Nadu, India; b Solid State and Structural Chemistry Unit, Indian Institute of Science, Bangalore 560 012, Karnataka, India; c Department of Physics, Faculty of Arts and Sciences, Erciyes University, 38039 Kayseri, Turkey

## Abstract

The title mol­ecule, C_5_H_7_N_3_O_2_, has an almost planar conformation, with a maximum deviation of 0.043 (3) Å, except for the methyl H atoms. In the crystal structure, inter­molecular C—H⋯O hydrogen bonds link the mol­ecules into layers parallel to the *bc* plane. Inter­molecular π–π stacking inter­actions [centroid–centroid distances = 3.685 (2) and 3.697 (2) Å] are observed between the parallel triazole rings.

## Related literature

For related structures, see: Prabakaran *et al.* (2009*a* ▶,*b* ▶); Beitelman *et al.* (2007 ▶); Jabli *et al.* (2010 ▶). For the properties and applications of related compounds, see: Dehne (1994 ▶); Fan & Katritzky (1996 ▶); Genin *et al.* (2000 ▶); Velazquez *et al.* (1998 ▶).
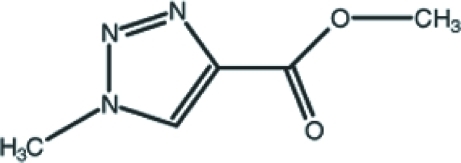


## Experimental

### 

#### Crystal data

C_5_H_7_N_3_O_2_
*M* *_r_* = 141.14
Triclinic, 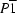

*a* = 5.697 (1) Å
*b* = 7.1314 (11) Å
*c* = 8.6825 (16) Å
α = 71.053 (16)°
β = 86.865 (15)°
γ = 76.528 (14)°
*V* = 324.37 (10) Å^3^
*Z* = 2
Mo *K*α radiation
μ = 0.11 mm^−1^
*T* = 293 K
0.15 × 0.10 × 0.05 mm

#### Data collection

Oxford Diffraction Xcalibur Eos (Nova) CCD detector diffractometer
6915 measured reflections
1108 independent reflections
800 reflections with *I* > 2σ(*I*)
*R*_int_ = 0.049

#### Refinement

*R*[*F* ^2^ > 2σ(*F* ^2^)] = 0.064
*wR*(*F* ^2^) = 0.173
*S* = 1.13
1108 reflections
93 parameters
H-atom parameters constrained
Δρ_max_ = 0.26 e Å^−3^
Δρ_min_ = −0.23 e Å^−3^

### 

Data collection: *CrysAlis PRO CCD* (Oxford Diffraction, 2009 ▶); cell refinement: *CrysAlis PRO CCD*; data reduction: *CrysAlis PRO RED* (Oxford Diffraction, 2009 ▶); program(s) used to solve structure: *SHELXS97* (Sheldrick, 2008 ▶); program(s) used to refine structure: *SHELXL97* (Sheldrick, 2008 ▶); molecular graphics: *ORTEP-3 for Windows* (Farrugia, 1997 ▶); software used to prepare material for publication: *WinGX* (Farrugia, 1999 ▶).

## Supplementary Material

Crystal structure: contains datablocks global, I. DOI: 10.1107/S1600536810019173/xu2764sup1.cif

Structure factors: contains datablocks I. DOI: 10.1107/S1600536810019173/xu2764Isup2.hkl

Additional supplementary materials:  crystallographic information; 3D view; checkCIF report

## Figures and Tables

**Table 1:** Hydrogen-bond geometry (Å, °)

*D*—H⋯*A*	*D*—H	H⋯*A*	*D*⋯*A*	*D*—H⋯*A*
C1—H1*A*⋯O1^i^	0.96	2.59	3.509 (5)	160
C5—H5*B*⋯O1^ii^	0.96	2.39	3.277 (5)	153

Symmetry codes: (i) 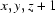; (ii) 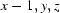.

**Fig. 1.**
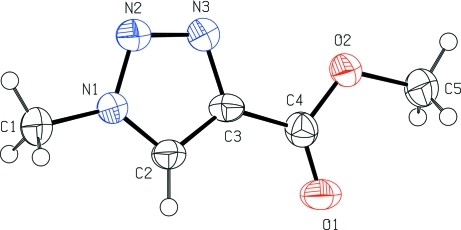
The title molecule showing the atom-numbering scheme. The displacement ellipsoids are drawn at the 50% probability level.

**Fig. 2.**
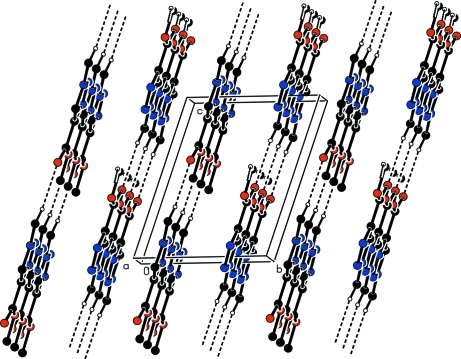
View of the crystal packing of (I), viewed down the a axis. The H atoms not involved in the hydrogen bonding pattern have been omitted for clarity.

## supplementary crystallographic information

### Comment

1,2,3-Triazoles are useful synthetic targets in organic synthesis and are associated with biological properties such as antiviral, antibacterial, antiepileptic and antiallergic (Velazquez *et al.*, 1998; Genin *et al.*, 2000). They have also found applications as agrochemicals, dyes, hotographic materials, and in corrosion inhibition (Fan & Katritzky, 1996; Dehne, 1994). In continuous of our earlier reports (Prabakaran *et al.*, 2009a,b), here the crystal structure of the title compound is presented.

As shown in Fig. 1, the conformation of the title molecule is almost planar, with a maximum deviation of -0.043 (3) Å for O1, except the H atoms of two methyl groups.

In the crystal structure, molecules connect to each other, via the intermolecular C—H···O hydrogen bonds (Table 1, Fig. 2), into two-dimensional layers parallel to the *bc* plane, and intermolecular π–π stacking interactions [*Cg*1···*Cg*1(1 - *x*, -y, 2 - *z*) = 3.685 (2) Å and *Cg*1···*Cg*1(1 - *x*, 1 - *y*, 2 - *z*) = 3.697 (2) Å, where Cg1 is a centroid of the triazole ring] between the parallel triazole rings contribute to the stabilization of the structure.

### Experimental

To methyl 1*H*-1,2,3-triazole-4-carboxylate (2 g) in dry DMF (15 ml) maintained at 273 K in nitrogen atmosphere, was added K_2_CO_3_ (1.3 g), methyliodide (0.98 ml), the mixture was then stirred at 273 K for 1 h, allowed to warm to room temperature and stirred till completion of reaction, monitored by TLC. The reaction mixture on LCMS analysis showed three isomers well separated with their significant retention time and high purity. Three fractions were identified by mass spectroscopy. The solvent was evaporated under vacuo and the residue was isolated into individual isomers by column chromatography. The single crystals of the title compound for X-ray structure analysis were obtained from ether solution by slow evaporation.

### Refinement

H atoms were positioned geometrically with C—H = 0.93-0.96 Å, and were refined in riding mode with U_iso_(H) = 1.2 or 1.5U_eq_(C). In the final refinement cycles, the inconsistent 33 reflections were omitted.

### Crystal data

**Table:**

C_5_H_7_N_3_O_2_	*Z* = 2
*M**_r_* = 141.14	*F*(000) = 148
Triclinic, *P*1	*D*_x_ = 1.445 Mg m^−^^3^
Hall symbol: -P 1	Mo *K*α radiation, λ = 0.71073 Å
*a* = 5.697 (1) Å	Cell parameters from 1326 reflections
*b* = 7.1314 (11) Å	θ = 2.0–20.7°
*c* = 8.6825 (16) Å	µ = 0.11 mm^−^^1^
α = 71.053 (16)°	*T* = 293 K
β = 86.865 (15)°	Block, colourless
γ = 76.528 (14)°	0.15 × 0.10 × 0.05 mm
*V* = 324.37 (10) Å^3^	

### Data collection

**Table:**

Oxford Xcalibur Eos (Nova) CCD detector diffractometer	800 reflections with *I* > 2σ(*I*)
Radiation source: Enhance (Mo) X-ray Source	*R*_int_ = 0.049
graphite	θ_max_ = 25.0°, θ_min_ = 3.1°
ω scans	*h* = −6→6
6915 measured reflections	*k* = −8→8
1108 independent reflections	*l* = −10→10

### Refinement

**Table:**

Refinement on *F*^2^	Primary atom site location: structure-invariant direct methods
Least-squares matrix: full	Secondary atom site location: difference Fourier map
*R*[*F*^2^ > 2σ(*F*^2^)] = 0.064	Hydrogen site location: inferred from neighbouring sites
*wR*(*F*^2^) = 0.173	H-atom parameters constrained
*S* = 1.13	*w* = 1/[σ^2^(*F*_o_^2^) + (0.0741*P*)^2^ + 0.2349*P*] where *P* = (*F*_o_^2^ + 2*F*_c_^2^)/3
1108 reflections	(Δ/σ)_max_ < 0.001
93 parameters	Δρ_max_ = 0.26 e Å^−^^3^
0 restraints	Δρ_min_ = −0.23 e Å^−^^3^

### Special details

**Table:**

Geometry. Bond distances, angles etc. have been calculated using the rounded fractional coordinates. All su's are estimated from the variances of the (full) variance-covariance matrix. The cell esds are taken into account in the estimation of distances, angles and torsion angles
Refinement. Refinement on *F*^2^ for ALL reflections except those flagged by the user for potential systematic errors. Weighted *R*-factors wR and all goodnesses of fit *S* are based on *F*^2^, conventional *R*-factors *R* are based on *F*, with *F* set to zero for negative *F*^2^. The observed criterion of *F*^2^ > σ(*F*^2^) is used only for calculating -*R*-factor-obs etc. and is not relevant to the choice of reflections for refinement. *R*-factors based on *F*^2^ are statistically about twice as large as those based on *F*, and *R*-factors based on ALL data will be even larger.

### Fractional atomic coordinates and isotropic or equivalent isotropic displacement parameters (Å^2^)

**Table:**

	*x*	*y*	*z*	*U*_iso_*/*U*_eq_	
O1	0.6694 (5)	0.2352 (5)	0.5832 (3)	0.0591 (13)	
O2	0.2797 (4)	0.2639 (4)	0.6475 (3)	0.0440 (9)	
N1	0.7108 (5)	0.2390 (4)	1.0644 (3)	0.0361 (10)	
N2	0.4740 (5)	0.2478 (5)	1.0897 (4)	0.0430 (11)	
N3	0.3757 (5)	0.2534 (5)	0.9553 (4)	0.0417 (10)	
C1	0.8687 (7)	0.2325 (6)	1.1941 (5)	0.0471 (14)	
C2	0.7651 (6)	0.2415 (5)	0.9131 (4)	0.0369 (11)	
C3	0.5521 (6)	0.2492 (5)	0.8434 (4)	0.0342 (11)	
C4	0.5106 (6)	0.2486 (5)	0.6793 (4)	0.0373 (12)	
C5	0.2257 (7)	0.2676 (7)	0.4852 (5)	0.0532 (14)	
H1A	0.77460	0.24230	1.28750	0.0700*	
H1B	0.94680	0.34440	1.15670	0.0700*	
H1C	0.98850	0.10660	1.22340	0.0700*	
H2	0.91520	0.23860	0.86500	0.0440*	
H5A	0.26750	0.38410	0.40620	0.0790*	
H5B	0.05650	0.27540	0.47490	0.0790*	
H5C	0.31730	0.14580	0.46690	0.0790*	

### Atomic displacement parameters (Å^2^)

**Table:**

	*U*^11^	*U*^22^	*U*^33^	*U*^12^	*U*^13^	*U*^23^
O1	0.0354 (15)	0.103 (3)	0.0513 (18)	−0.0203 (15)	0.0119 (13)	−0.0399 (17)
O2	0.0336 (14)	0.0667 (18)	0.0370 (15)	−0.0139 (12)	−0.0013 (11)	−0.0216 (13)
N1	0.0315 (16)	0.0468 (19)	0.0314 (17)	−0.0104 (13)	0.0007 (13)	−0.0133 (13)
N2	0.0346 (17)	0.057 (2)	0.0386 (18)	−0.0098 (14)	0.0038 (14)	−0.0178 (15)
N3	0.0348 (16)	0.055 (2)	0.0354 (18)	−0.0108 (14)	0.0002 (14)	−0.0140 (14)
C1	0.045 (2)	0.062 (3)	0.036 (2)	−0.0133 (19)	−0.0046 (17)	−0.0165 (19)
C2	0.0306 (18)	0.047 (2)	0.036 (2)	−0.0126 (16)	0.0054 (15)	−0.0155 (17)
C3	0.0286 (18)	0.038 (2)	0.039 (2)	−0.0116 (15)	0.0068 (15)	−0.0144 (16)
C4	0.038 (2)	0.039 (2)	0.038 (2)	−0.0120 (16)	−0.0022 (18)	−0.0137 (17)
C5	0.046 (2)	0.082 (3)	0.040 (2)	−0.025 (2)	−0.0030 (18)	−0.023 (2)

### Geometric parameters (Å, °)

**Table:**

O1—C4	1.205 (4)	C3—C4	1.459 (5)
O2—C4	1.331 (4)	C1—H1A	0.9600
O2—C5	1.449 (5)	C1—H1B	0.9600
N1—N2	1.345 (4)	C1—H1C	0.9600
N1—C1	1.462 (5)	C2—H2	0.9300
N1—C2	1.328 (4)	C5—H5A	0.9600
N2—N3	1.307 (5)	C5—H5B	0.9600
N3—C3	1.361 (5)	C5—H5C	0.9600
C2—C3	1.367 (5)		
			
O1···C5^i^	3.277 (5)	C4···N2^vii^	3.439 (5)
O2···N3	2.731 (4)	C5···C1^x^	3.435 (6)
O1···H1A^ii^	2.5900	C5···O1^vi^	3.277 (5)
O1···H5A	2.6300	C1···H5B^ix^	2.8500
O1···H5B^i^	2.3900	C4···H5A^iv^	3.0300
O1···H5C	2.5900	H1A···O1^xi^	2.5900
O1···H1C^iii^	2.8400	H1A···H5B^ix^	2.4500
O1···H5A^iv^	2.8500	H1B···N3^viii^	2.9100
O1···H5C^v^	2.8700	H1C···O1^iii^	2.8400
O1···H2	2.8900	H2···O1	2.8900
O2···H2^vi^	2.7300	H2···O2^i^	2.7300
N2···C3^vii^	3.438 (5)	H2···N3^i^	2.8100
N2···C4^vii^	3.439 (5)	H5A···O1	2.6300
N3···O2	2.731 (4)	H5A···O1^iv^	2.8500
N3···C1^viii^	3.434 (6)	H5A···C4^iv^	3.0300
N3···C3^vii^	3.376 (5)	H5B···O1^vi^	2.3900
N3···H2^vi^	2.8100	H5B···C1^x^	2.8500
N3···H1B^viii^	2.9100	H5B···H1A^x^	2.4500
C1···C5^ix^	3.435 (6)	H5C···O1	2.5900
C1···N3^viii^	3.434 (6)	H5C···O1^v^	2.8700
C3···N3^vii^	3.376 (5)	H5C···H5C^v^	2.5200
C3···N2^vii^	3.438 (5)		
			
C4—O2—C5	115.6 (3)	N1—C1—H1B	109.00
N2—N1—C1	120.4 (3)	N1—C1—H1C	110.00
N2—N1—C2	110.7 (3)	H1A—C1—H1B	109.00
C1—N1—C2	129.0 (3)	H1A—C1—H1C	109.00
N1—N2—N3	107.9 (3)	H1B—C1—H1C	109.00
N2—N3—C3	107.9 (3)	N1—C2—H2	128.00
N1—C2—C3	105.0 (3)	C3—C2—H2	127.00
N3—C3—C2	108.6 (3)	O2—C5—H5A	109.00
N3—C3—C4	123.5 (3)	O2—C5—H5B	109.00
C2—C3—C4	127.9 (3)	O2—C5—H5C	110.00
O1—C4—O2	123.8 (3)	H5A—C5—H5B	109.00
O1—C4—C3	123.3 (3)	H5A—C5—H5C	109.00
O2—C4—C3	112.9 (3)	H5B—C5—H5C	109.00
N1—C1—H1A	109.00		
			
C5—O2—C4—O1	−1.1 (6)	N2—N3—C3—C4	178.5 (3)
C5—O2—C4—C3	179.0 (3)	N1—C2—C3—N3	0.7 (4)
C1—N1—N2—N3	179.6 (3)	N1—C2—C3—C4	−178.0 (3)
C2—N1—N2—N3	0.8 (4)	N3—C3—C4—O1	−176.4 (4)
N2—N1—C2—C3	−0.9 (4)	N3—C3—C4—O2	3.5 (5)
C1—N1—C2—C3	−179.6 (4)	C2—C3—C4—O1	2.1 (6)
N1—N2—N3—C3	−0.3 (4)	C2—C3—C4—O2	−178.0 (4)
N2—N3—C3—C2	−0.2 (4)		

Symmetry codes: (i) *x*+1, *y*, *z*; (ii) *x*, *y*, *z*−1; (iii) −*x*+2, −*y*, −*z*+2; (iv) −*x*+1, −*y*+1, −*z*+1; (v) −*x*+1, −*y*, −*z*+1; (vi) *x*−1, *y*, *z*; (vii) −*x*+1, −*y*, −*z*+2; (viii) −*x*+1, −*y*+1, −*z*+2; (ix) *x*+1, *y*, *z*+1; (x) *x*−1, *y*, *z*−1; (xi) *x*, *y*, *z*+1.

### Hydrogen-bond geometry (Å, °)

**Table:**

*D*—H···*A*	*D*—H	H···*A*	*D*···*A*	*D*—H···*A*
C1—H1A···O1^xi^	0.96	2.59	3.509 (5)	160
C5—H5B···O1^vi^	0.96	2.39	3.277 (5)	153

Symmetry codes: (xi) *x*, *y*, *z*+1; (vi) *x*−1, *y*, *z*.
